# Comparative transcriptomic and metabolomic analyses reveal the delaying effect of naringin on postharvest decay in citrus fruit

**DOI:** 10.3389/fpls.2022.1045857

**Published:** 2022-11-30

**Authors:** Jiaoke Zeng, Chuying Chen, Ming Chen, Jinyin Chen

**Affiliations:** Jiangxi Key Laboratory for Postharvest Technology and Nondestructive Testing of Fruits & Vegetables, College of Agronomy, Jiangxi Agricultural University, Nanchang, China

**Keywords:** citrus fruit, naringin, metabolomics, transcriptomics, decay, antioxidant

## Abstract

**Introduction:**

Naringin exhibits antioxidant capacity and can partially inhibit pathogens in many horticultural products, such as citrus fruit; however, the effects of naringin on the storage quality and mechanisms that regulate senescence in citrus fruit have not been comprehensively analyzed.

**Methods and results:**

In this study, exogenous naringin treatment was found to significantly delay citrus fruit disease, decreasing the H_2_O_2_ content, increasing the antioxidant capacity and maintaining the quality of the fruit. Metabolomic analysis of citrus peel indicated the vast majority (325) of metabolites belonging to flavonoids. Moreover, the auraptene, butin, naringenin, and luteolin derivative levels within the phenylpropanoid pathway were significantly higher in the naringin-treated fruit than in the control fruit. Transcriptomic analysis also revealed that twelve genes in the phenylpropanoid and flavonoid biosynthesis pathways were significantly upregulated. Further analysis with a co-expression network revealed significant correlation between these differential genes and metabolites. Additionally, MYC and WRKY, screened from the MAPK signaling pathway, may contribute to naringin-induced disease resistance.

**Conclusion:**

In conclusion, naringin treatment can efficiently delay decay and maintain the quality of citrus fruit, mainly by promoting metabolites accumulation, and upregulating differentially expressed genes in phenylpropanoid and flavonoid biosynthesis pathway. This study provides a better understanding of the regulatory mechanisms through which naringin delays citrus fruit decay and maintains fruit quality.

## Introduction

Citrus (*Citrus reticulata* Blanco.) fruit, arguably one of the most important cultivated fruit worldwide, is highly desired by consumers because of its bright appearance, pleasant taste, and abundance of health-promoting compounds (e.g., phenols and flavonoids) (Singh et al., 2020). As typical non-climacteric fruits, most citrus fruits, such as mandarins and oranges, are harvested at the fully ripe stage and can have extended shelf life under suitable conditions (Gao et al., 2019; Kumar et al., 2022). However, such fruits are susceptible to pathogens; pathogenic infection can result in quality deterioration and decay during postharvest storage (Berk, 2016). Various treatments have been applied to treat citrus before storage, such as temperature control (Gao et al., 2019), coatings (Gao et al., 2018), phytohormones (Ma et al., 2014), antioxidants (Sdiri et al., 2014; Lin et al., 2021), antagonistic microorganisms (Zhou et al., 2018b; Chen et al., 2021), and other inhibitors of pathogenic microorganisms (Duan et al., 2021). Nevertheless, undesirable residues and relatively low disease resistance can still affect citrus fruits during postharvest handling. Thus, safer and more efficient applications, such as natural products derived from plants that can reduce citrus decay, maintain quality attributes, and prolong shelf life have been widely explored.

Flavonoids are plant secondary metabolites that include flavanols, flavanones, flavones, flavonols, isoflavones, and anthocyanins (Panche et al., 2016). They are widely found in several fruits, vegetables, and beverages, and have miscellaneous bioactive functions, including anti-oxidative, anti-inflammatory, and anti-carcinogenic properties that contribute to disease resistance (Yao et al., 2004; Zaidun et al., 2018; Gupta et al., 2022). The biosynthesis and regulation of flavonoids have been widely studied in plants (Liu et al., 2021). These compounds may participate in plant development, abiotic stress, pathogen defense, biosynthesis, and metabolite accumulation (Winkel-Shirley, 2002; Treutter, 2005; Sharma et al., 2019). More than 80 flavonoids found in citrus fruits can be classified flavanones, flavones, flavonols, and anthocyanins (Tripoli et al., 2007; Xi et al., 2014). Moreover, several genes that are related to the biosynthesis of the citrus flavonoid skeleton have been identified *via* molecular technology and genomics strategies in recent decades, including those that encode chalcone isomerase (CHI), flavone synthase (FNS), flavone 3-hydroxylase (F3H), flavonoid 3’-hydroxylase (F3’H), flavonol synthase (FLS), and UDP-dependent glycosyltransferases (UGTs). (Zhao et al., 2020). Nevertheless, the roles of flavonoids in plant resistance and stress, as well as the regulatory mechanisms that control their biosynthesis in postharvest fruit, remain to be further investigated.

Previous studies have reported the role of flavonoids (polymethoxyflavones, naringin, hesperidin, and neohesperidin) in disease resistance, providing insight into the antifungal resistance of citrus fruits (Ortuño et al., 2006; Salas et al., 2011). Moreover, as antioxidants, flavonoids also play important roles in maintaining postharvest quality and delaying senescence in fruits and vegetables by enhancing antioxidative systems, increasing gene expression levels and the activities of antioxidant enzymes, promoting the content of antioxidant compounds, and scavenging reactive oxygen species (Cavia-Saiz et al., 2010; Roghini and Vijayalakshmi, 2018; Meitha et al., 2020). Studies on litchi and banana fruit have reported that procyanidins, which are classified as flavanols, maintain quality and delay senescence by promoting antioxidant activity and enhancing endogenous flavonoid biosynthesis (Chen et al., 2019; Bai et al., 2022). Anthocyanins, colorful pigments that belong to the flavonoid subgroup, contribute to the defense against postharvest green mold (Lin et al., 2021). Other flavonoids, such as flavones, flavanones, and polymethoxyflavones, also engage in the defense mechanism of citrus fruits against *P. digitatum* and *P. italicum* (Ortuño and Río, 2009; Ortuño et al., 2011). However, the effects and mechanisms by which various flavonoids affect postharvest citrus fruit preservation require further research.

In the present study, comparative transcriptomics and metabolomics were conducted to explore the fundamental regulatory pathways of naringin in maintaining Nanfeng mandarin fruit quality and delaying disease occurrence. Our results provide insight into fungal disease defense in citrus fruits after treatment with naringin and other flavonoids.

## Materials and methods

### Plant materials and treatment

Citrus (*Citrus reticulata* Blanco. cv. ‘Nanfeng mandarin’) fruits were harvested at commercial maturity from a citrus orchard in Nanfeng City, Jiangxi Province, China in 2021. Fruit of uniform size and color with no mechanical injury or disease was collected and transferred to the laboratory within 3 h. Fruit sample was randomly divided into two groups of approximately 600 individuals. The groups were soaked for 5 min in 5 g/L naringin (food-grade) or tap water (Control) respectively. The fruit was then air-dried, packed into polyethylene bags (0.05 mm thickness), and stored at room temperature for 60 d. The decay rate, fruit quality, and antioxidant capacity were examined every ten d of storage. Peel tissues were sampled according to the results of decay and antioxidant properties and delivered for metabolomic and transcriptomic sequencing. Three biological replicates of ten fruit each were examined at each interval, and another 300 fruit samples for each treatment were included to examine weight loss and decay rate.

### Fruit quality analysis

Fruit quality and physiology were determined according to previous methods (Chen et al., 2016). Citrus decay rate was visually evaluated and expressed as the percentage of fruit showing infection of disease (Chen et al., 2016). Weight loss was calculated by comparison with the initial weight every ten d (Chen et al., 2016). Respiration rate was measured using a respiration-detecting device (JFQ-3150H, Jun-fang-li-hua Tech. China). Fruit from each replicate was placed in a sealed chamber at room temperature, after which any changes in CO_2_ concentration were recorded within 5 min. The respiration rate was expressed as mgCO_2_/kg(FW)/h. Peel color was measured using a chroma meter (CR-400, Konica Minolta, Inc.), and color changes were quantified *via* the L*, a*, and b* values. Total soluble solids (TSS, %) were measured using a refractometer (ATAGO, Tokyo, Japan). Total acid (TA, %) was monitored using a titration of citrus juice with sodium hydroxide and was expressed as % citric acid (Cao et al., 2011). Vitamin C (VC) was measured using the 2,6-dichlorophenol-indophenol titrimetric method described by Kampfenkel et al. (1995), with the content expressed as mg/100 g FW of pulp. All detections were conducted three times.

### Analysis of antioxidant properties

The total phenol and flavonoid contents were determined using methods in Chen et al. (2016), with slight modifications. Frozen peel (0.5 g) powder was added to 8 mL of 1% (v/v) ice-cold HCl-methanol solution and centrifuged at 12,000 × *g* and 4°C for 15 min. The supernatant was then collected and used to detect absorbance at 280 nm and 325 nm. The total phenols and flavonoids were expressed as OD_280_/g FW and OD_325_/g FW, respectively.

The total antioxidant capacity was measured according to the method described by Cao et al. (2011). Citrus peel powder (2 g) was dissolved in 8 mL of absolute ethanol, centrifuged at 12,000 rpm at 4°C for 20 min, and the supernatant (100 μL) was collected and mixed with 2.9 mL DPPH in methanol (0.2 mM). The reaction mixture was incubated at room temperature for 30 min and the absorbance at 517 nm recorded. Total antioxidant capacity was evaluated as the DPPH radical scavenging rate (%).

### Determinations of hydrogen peroxide content and enzymes activities

The hydrogen peroxide (H_2_O_2_) content was determined by applying an H_2_O_2_ detection kit (article number: BC3595, Beijing Solarbio & Technology CO., Ltd, Beijing, China). Frozen peel powder (0.1 g) was dissolved in 1 mL of ice-cold acetone, centrifuged at 8,000 × *g* at 4°C for 10 min, and the supernatant (250 μL) was collected and detected according to the manufacturer’s instructions. H_2_O_2_ content was expressed as μmol/g FW.

Phenylalnine ammonialyase (PAL; EC.4.3.1.5) and Peroxidase (POD; EC 1.11.1.7) activity were determined using detection kits (article number: BC0215 and BC0095, Beijing Solarbio & Technology CO., Ltd, Beijing, China). One unit (U) of PAL activity was defined as the amount of enzyme that causes an increase of 0.05 in absorbance at 290 nm. One U of POD activity was defined as an increase of 1 in absorbance at 470 nm.

Catalase (CAT; EC 1.11.1.6) and Polyphenol oxidase (PPO; EC 1.10.3.2) activities were measured according to the method in Cao et al. (2011). 0.5 g frozen peel was dissolved in 5 mL of 0.1 M phosphate buffer (pH 7.5) containing 5 mM DTT and 5% (*m*/*v*) PVP. After centrifugation at 12,000 × *g* at 4°C for 30 min, the supernatant was obtained and used to detect the CAT activity. One unit of CAT activity was defined as a decrease of 0.01 in absorbance at 240 nm. Frozen peel (0.5 g) powder was added to 5 mL of acetic acid buffer (pH 5.5) containing 1 mM PEG, 4% (*m*/*v*) PVPP, and 1% Triton X-100. The supernatant was collected by centrifugation at 12,000 × *g* at 4°C for 30 min and used to detect PPO activity. An increase of 1 in absorbance at 420 nm was defined as one U of PPO activity. PAL, POD, CAT, and PPO activities were expressed as U/min/g FW.

### Metabolome analysis

Based on the browning and antioxidant properties, three biological samples were collected from fresh-harvested (0 DAH), fruit subjected to controlled storage for 40 d (CK_40 DAH), and naringin-treated peels (Nar_40 DAH). A UPLC-ESI-MS/MS system with a SB-C18 column (1.8 µm, 2.1 mm × 100 mm, Agilent) connected to an ESI- QTRAP 4500 (AB Sciex) was used for metabolome analysis (Metware, Wuhan, China). In the present study, the mobile phase consisted of solvents: (A) 0.1% formic acid in pure water, and (B) 0.1% formic acid in acetonitrile. The elution gradient program was initiated with 95% A and 5% B, followed by a linear gradient to 5% A and 95% B within 9 min, 5% A and 95% B over 1 min, 95% A and 5.0% B for 1.1 min, and maintained for 2.9 min. A 0.35 mL/min flow rate and an injection volume of 4 μL were used. MS/MS was performed in QQQ and LIT mode using Analyst 1.6.3 software (AB Sciex). The ESI source parameters: ion source gas I and II were set at 50 and 60 psi, curtain gas pressure was 25.0 psi, ion spray voltage (IS) was 5500 V (positive ion mode) or -4500V (negative ion mode), respectively, were used with a source temperature of 550°C.

MS data were processed using the R package software (version 3.5) (Chong and Xia, 2018). An MS2 database was used for metabolite identification (Chen et al., 2013). Orthogonal projection to latent structures discriminant analysis (OPLS-DA) and principal component analysis (PCA) were conducted to analyze the differences and reliability of the metabolites in the peels (Eriksson et al., 2006). Significantly differential metabolites (DMs) in each group were determined by variable importance in projection (VIP) ≥1 and absolute log_2_FC ≥1. The DMs were then annotated to the KEGG compound database and p-values were used to determine significance.

### cDNA library construction and RNA sequencing

Freshly harvested, control, and naringin-treated citrus peels were collected at day 40 for RNA sequencing. Total RNA was extracted using the TRIzol reagent kit (Invitrogen, Carlsbad, USA) according to the manufacturer’s protocol. RNA quality was evaluated with an Agilent 2100 Bioanalyzer (Agilent Technologies, Palo Alto, USA) and agarose gel electrophoresis. The mRNA was then enriched using Oligo (dT) beads. First-strand cDNA was synthesized with random primers, and second-strand cDNA was synthesized using DNA polymerase I, RNase H, dNTP, and buffer. Double-stranded cDNA fragments were purified using the QiaQuick PCR extraction kit (Qiagen, Venlo, Netherlands). End-repaired and poly (A) were added to the sequencing adapters before ligation. Finally, the ligation products were PCR-amplified and sequenced using an Illumina HiSeq 2500 by Gene Denovo Biotechnology Co. (Guangzhou, China). Clean reads were obtained by filtering the raw reads containing adapters or low-quality bases using fastp (version 0.18.0). Paired-end clean reads were mapped to the citrus genome database (https://www.citrusgenomedb.org/organism/Citrus/reticulata) by HISAT2.2.4 (Kim et al., 2015).

### Functional annotation and differential genes screening

Gene expression abundance was quantified using the StringTie software and expressed as an FPKM value. Differentially expressed genes (DEGs) in the two groups were identified using DESeq2 (Love et al., 2014). DEGs with an absolute fold change > 2 and a false discovery rate (FDR) < 0.05 were screened. Finally, the R package gmodels (http://www.r-project.org/) was used for PCA, and the GO (Ashburner et al., 2000) and KEGG (Kanehisa and Goto, 2000) databases were used for biological functional analysis of the DEGs.

### Quantitative RT-PCR validation of RNA-seq data

The expression patterns of 12 DEGs involved in flavonoid biosynthesis and the MAPK signaling pathway were validated using quantitative real-time PCR (qRT-PCR) with gene-specific primers designed using Primer 3 (http://frodo.wi.mit.edu/primer3/), which are listed in **Supplementary Table S1**. Melting curves and qRT-PCR product re-sequencing were used to check the quality and specificity of the primers. Total RNA was extracted from citrus peels as described above. cDNA was synthesized using the Hifair^®^ III 1^st^ Strand cDNA Synthesis SuperMix for qPCR (gDNA digester plus) (Yeasen Biotechnology Co., Ltd. Shanghai, China). qRT-PCR was performed on a CFX96™ Real-Time System (Bio-Rad). The procedure was initiated for 30 s at 95°C, followed by 40 cycles of 5 s at 95°C and 30 s at 60°C. Melting curve analysis was performed at 95°C for 15s, 60°C for 30s, and 95°C for 5s. The PCR mixture (10 μL) contained 5 μL of Hieff^®^ qPCR SYBR Green Master Mix (No Rox), 0.3 μL of each primer (10 μM), 1 μL of cDNA, and 3.4 μL of sterile H_2_O. Relative gene expression was calculated using 0 DAH fruit for calibration, and *CsActin* was used as the internal reference gene to normalize transcript levels following the 2^-△△Ct^ method. Three biological replicates were used in each experiment.

### Statistical analysis

All data were analyzed using SPSS 26.0. Data are expressed as the mean ± standard error (SE) of the triplicates. The variance (PROC ANOVA) was further analyzed with multi-comparison correction, followed by Duncan’s multiple range test at the 5% level. Origin 8.0 (Microcal Software Inc., Northampton, MA, USA) was used to construct the figures.

## Results

### Analysis of fruit quality

**Figure 1:**
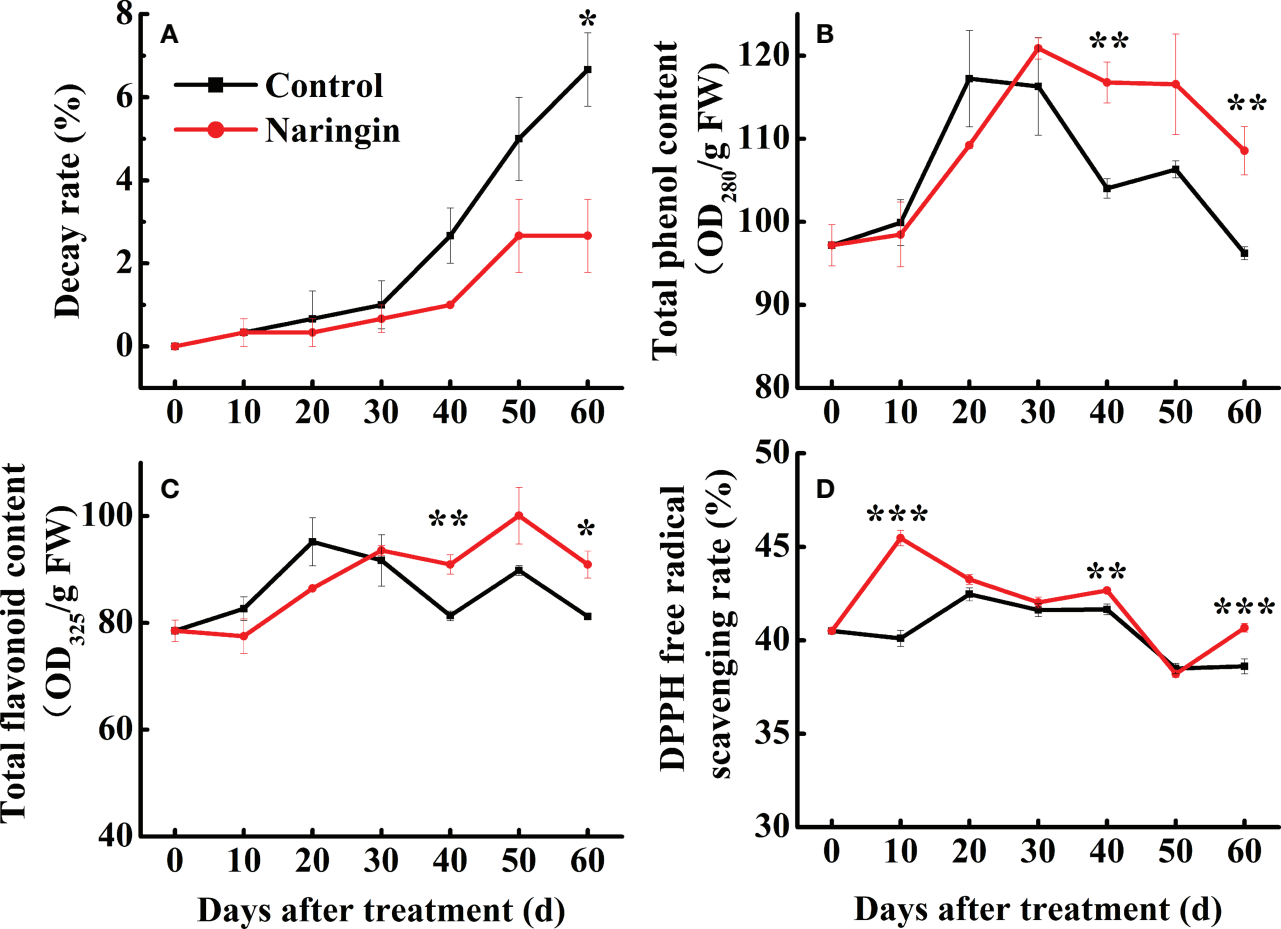
The effects of naringin treatment on decay rate **(A)**, total phenol content **(B)**, total flavonoid content **(C)**, and DPPH free radical scavenging rate **(D)** of Nanfeng mandarin fruit during room temperature storage. The asterisk of * (*P* < 0.05), ** (*P* < 0.01) or *** (*P* < 0.001) within the same day denotes a significant difference between control and naringin treatment.

Gradual decay was observed in both the control and the naringin-treated fruits during the first 30 d of storage. However, the rate of decay was significantly lower in citrus fruit treated with naringin than the controls (*P <*0.05) (**Figure 1A**). Fruit weight loss gradually increased during senescence, while the decrease trend were observed in the L* value, b* value, TA, and VC during the whole storage. In addition, fruit that were treated with naringin maintained relatively higher TSS and VC, and lower a* and b* levels, while the effects on attributes such as fruit respiration rate, weight loss, L*, and TA content were negligible (**Supplementary Table S2**).

### Analysis of antioxidant properties

As shown in **Figures 1B, C**, the total phenol and flavonoid content in both groups increased steadily and then peaked after 20 d of storage. Fruit treated with naringin maintained notably higher levels of total phenols and total flavonoids (*P <*0.05) than the control. The total antioxidant capacity increased slightly during the first 20 d of storage, which was followed by a relatively mild decrease thereafter (**Figure 1D**). Naringin treatment significantly increased the free radical scavenging rate (*P <*0.05), especially during the first 10 d, followed by a similar pattern to that observed for the control (**Figure 1D**). These results indicate that naringin treatment can increase antioxidant capacity by enhancing the total phenol and flavonoid contents.

### Analysis of H_2_O_2_ content and major antioxidant enzymes activities

**Figure 2:**
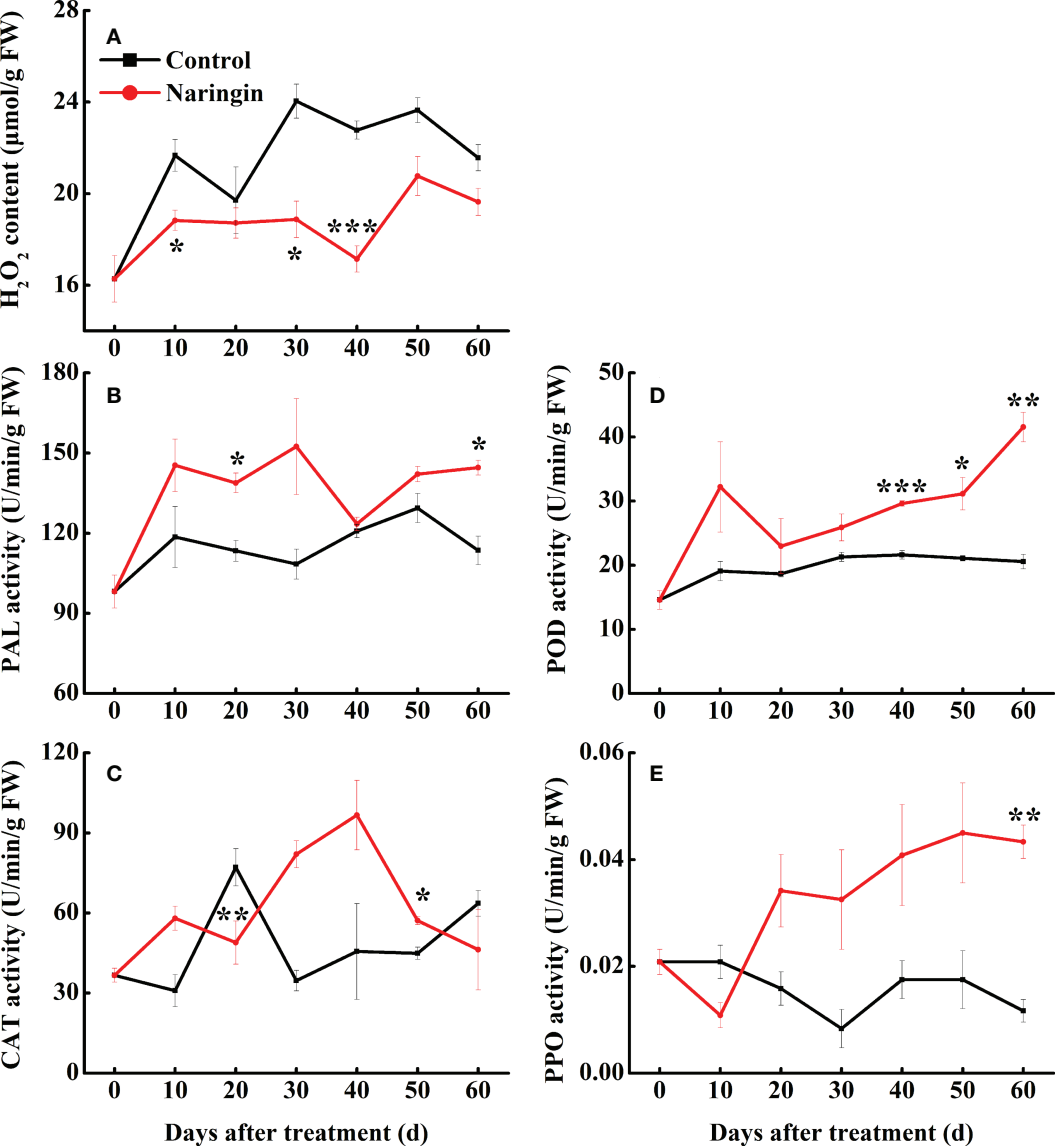
The effects of naringin treatment on H_2_O_2_ content **(A)**, PAL **(B)**, CAT **(C)**, POD **(D)**, and PPO **(E)** activities of Nanfeng mandarin fruit during room temperature storage. The asterisk of * (*P* < 0.05), ** (*P* < 0.01) or *** (*P* < 0.001) within the same day denotes a significant difference between control and naringin treatment.

As shown in **Figure 2A**, H_2_O_2_ content of the control peel fluctuating increase during the first 30 days of storage, but a slight decrease was observed thereafter. The H_2_O_2_ content was significantly lower (*P <*0.05) in the naringin-treated peels than in the control, especially in the middle storage period. PAL activity in control fruit increased slowly during storage, while the increase trends was enhanced after naringin treatment, especially in 20- and 60-d (**Figure 2B**). The activity of CAT in the control group peaked on day 20. After naringin treatment, the activity was higher than those of the control group, peaking on day 40 (**Figure 2C**). The activities of POD and PPO in control group changed gently, after naringin treatment, the activities were significantly higher than those of the control group after 30 days of storage (*P <*0.05) (**Figures 2D, E**).

### Profiling different citrus fruit metabolites during decay

**Figure 3:**
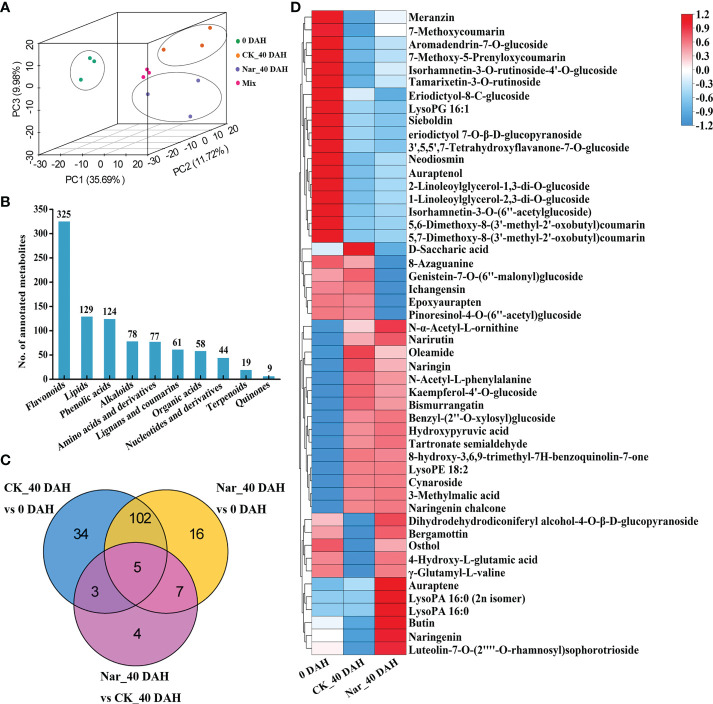
Analysis of DMs in citrus fruit peel. **(A)** PCA plot of the citrus peel after naringin treatment. Mix, quality control samples. **(B)** Number of all annotated metabolites in different categories. **(C)** Venn diagram of DMs. **(D)** Clustering heatmap of 50 DMs from CK_40 DAH vs 0 DAH and Nar_40 DAH vs 0 DAH.

Metabolite changes in the control and naringin-treated citrus peels were analyzed by UPLC-MS/MS to characterize the physiological processes that occur during decay. PCA analysis was performed to ascertain the next step of metabolite analysis (**Figure 3A**), with results confirming that the CK_40 DAH and Nar_40 DAH samples could be clearly differentiated from 0 DAH *via* PC1 (35.69%) (**Figure 3A**). A total of 1010 metabolites were detected by matching the MS spectra with the Metware database, and 994 were annotated using the KEGG compound database (**Supplementary Table S3**). The metabolites were divided into ten groups; 325 flavonoids, 129 lipids, 124 phenolic acids, 78 alkaloids, 77 amino acids and their derivatives, 61 lignans and coumarins, 58 organic acids, 44 nucleotides and their derivatives, 19 terpenoids, and 9 quinones (**Figure 3B**).

In all comparison groups, 171 metabolites appeared to differ, with a total of 5 metabolites exhibiting significant differences in each combination (**Figure 3C**). To study the crucial metabolic processes that are related to disease resistance in citrus peels, DMs for CK_40 DAH vs 0 DAH and Nar_40 DAH vs 0 DAH were screened. A total of 50 DMs were obtained and are presented in the heatmap, which shows the metabolite changes following Nar treatment as compared to the initial decay (**Figure 3D**). Of these, 18 and 15 metabolites were significantly down- or up-regulated, respectively, after 40 d of storage. Six DMs were downregulated and 11 were significantly upregulated as compared to 0 DAH and CK_40 DAH, with two lipids, one coumarin, and three flavonoids affected. These results indicate that flavonoids and coumarins in the phenylpropanoid pathway undergo significant change during decay, especially the Nar-treated group with mild disease symptoms.

### Transcriptomic analysis and annotation

**Figure 4:**
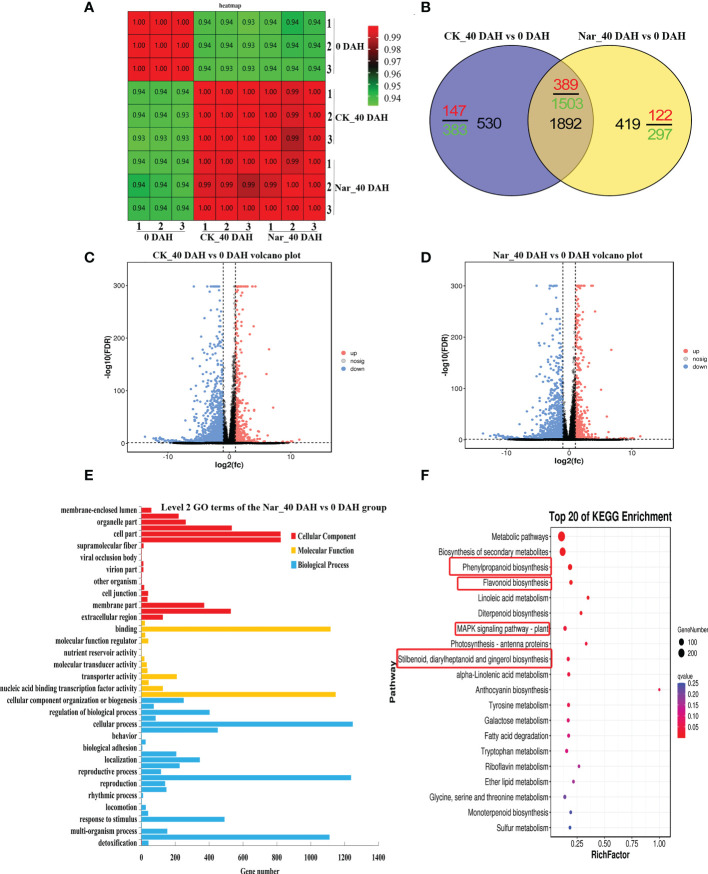
Preliminary analysis of transcriptomic data describing citrus fruit during storage at room temperature. **(A)** Correlation heatmap of different treatment groups. **(B)** Venn diagram of DEGs. **(C)** Volcano plots of DEGs in CK_40 DAH vs 0 DAH. **(D)** Volcano plots of DEGs in Nar_40 DAH vs 0 DAH. **(E)** GO functional classification of DEGs in the Nar_40 DAH vs 0 DAH group. **(F)** Significant enrichment KEGG analysis of DEGs in the Nar_40 DAH vs 0 DAH group. Highlighted red boxes are associated with Nar-induced disease resistance pathways.

Analysis of **Figure 1** indicates that naringin treatment significantly inhibits citrus decay and enhances antioxidant capacity after 30 d storage. Therefore, citrus peels fresh-harvested, 0 DAH peel from the control group was selected alongside peel from fruit that had been stored for 40 d in the naringin-treated group for transcriptome analysis. **Figure 4A** shows the correlation in the treatment and control groups, with correlation close to 1.000. These results reflect the significant difference between the treatment and control groups. **Figure 4B** shows that the DEGs in the CK_40 DAH vs 0 DAH and Nar_40 DAH vs 0 DAH groups overlapped. A total of 2422 and 2311 genes were differentially expressed in the CK_40 DAH and Nar_40 DAH groups after 40 d of storage, respectively, compared to the 0 DAH group, which revealed 1892 identical genes (**Supplementary Table S4**). These DEGs can be considered key regulators during the 40 d of storage.

Volcano maps were drawn to identify the differential gene distribution in the CK_40 DAH vs 0 DAH and Nar_40 DAH vs 0 DAH groups. In **Figures 4C, D**, blue dots indicate downregulated genes (Log_2_FC < -1), pink dots indicate upregulated genes (Log_2_FC > 1), and gray dots indicate insignificantly expressed genes (-1 < Log_2_FC < 1). The results show that there were 536 upregulated and 1886 downregulated genes in the CK_40 DAH vs 0 DAH group and 511 upregulated and 1800 downregulated genes in the Nar_40 DAH vs 0 DAH group, which indicates that a large number of DEGs were expressed during decay, and that the number of DEGs increased as the fruit decayed.

DEGs were analyzed using GO and KEGG analyses. As shown in **Figure 4E**, DEGs in the Nar_40 DAH vs 0 DAH group could be divided into biological processes, cellular components, and molecular functions, and were subdivided into 57 secondary functional groups. Of these, 25 subclasses are induced by biological processes, with cellular process and metabolic process the most abundant. The molecular functions comprised 13 subclasses, with catalytic activity and binding activity the most enriched, while 19 subclasses were observed in the cellular components, with cell parts the most abundant.

The DEGs were enriched in four pathways that are associated with disease resistance in the Nar_40 DAH vs 0 DAH group (**Figure 4F**); phenylpropanoid biosynthesis, flavonoid biosynthesis, stilbenoid, diarylheptanoid and gingerol biosynthesis, and the MAPK signaling pathway. Of these, 12 upregulated and 62 downregulated DEGs were observed in phenylpropanoid and flavonoid biosynthesis pathway (**Supplementary Figure S1**).

### Integrating related genes and metabolites in the phenylpropanoid pathway

**Figure 5:**
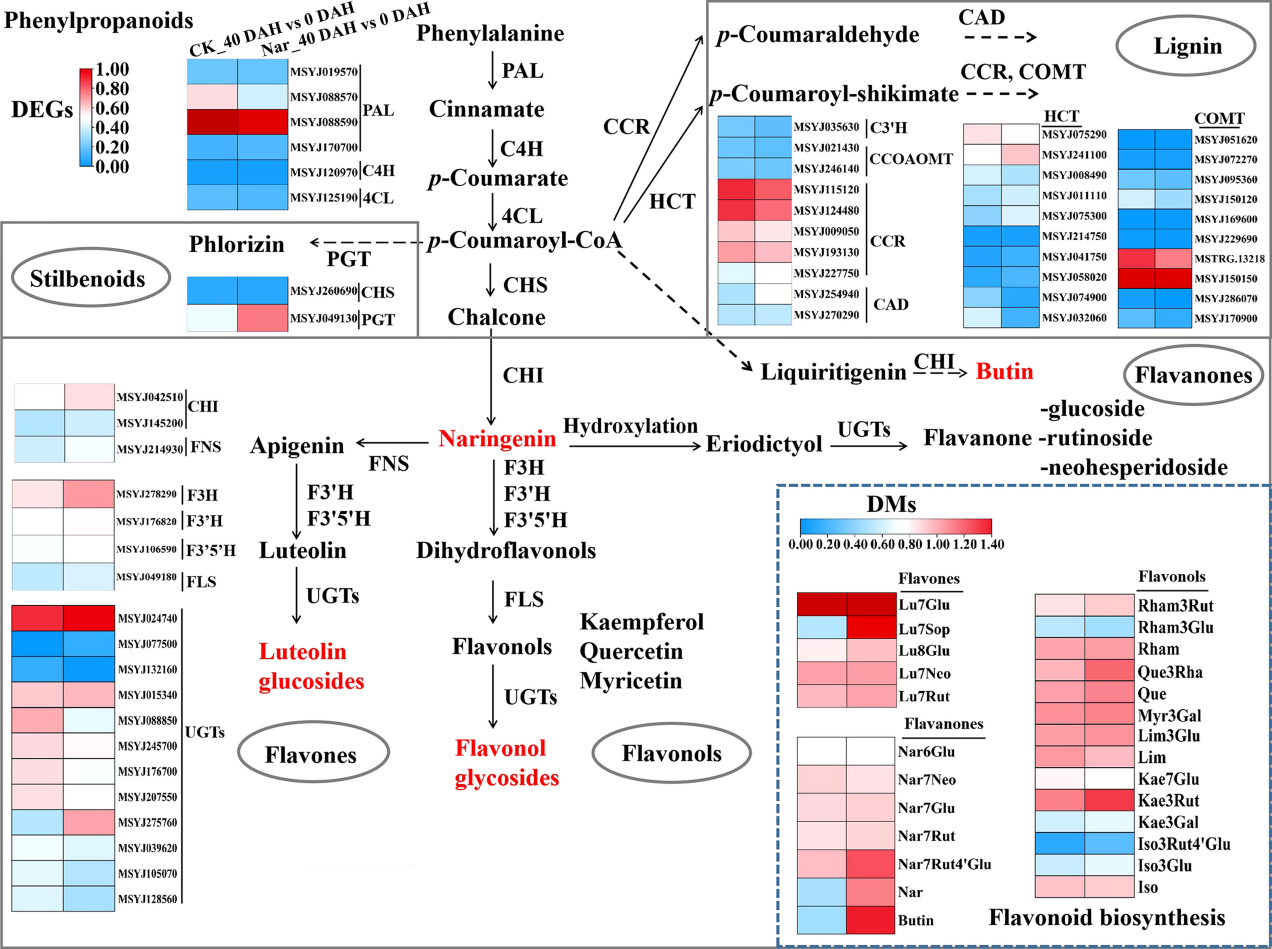
Diagram of phenylpropanoid and flavonoid biosynthesis pathways with their related DEGs and DMs. PAL, phenylalanine ammonia lyase; C4H, cinnamate 4-hydroxylase; 4CL, 4-coumarate:CoA ligase; CHS, chalcone synthase; CHI, chalcone isomerase; PGT, phlorizin synthase; C3H, coumarate 3-hydroxylase; HCT, shikimate O-hydroxycinnamoyltransferase; CCR, cinnamoyl CoA reductase; CAD, cinnamyl alcohol dehydrogenase; CCOAOMT, caffeoyl-CoA O-methyltransferase; COMT, caffeic acid 3-O-methyltransferase; FNS, flavone synthase; F3H, flavanone 3-hydroxylase; F3′H, flavonoid 3′-hydroxylase; F3′5′H, flavonoid 3′,5′-hydroxylase; FLS, flavonol synthase; UGTs, UDP-glycosyltransferase; Lu, luteolin; Nar, naringenin; Rham, Rhamnetin; Que, Quercetin; Myr, Myricetin; Lim, Limocitrin; Kae, Kaempfero; Iso, Isorhamnetin; Glu, glucoside; Neo, neohesperidoside; Rut, rutinoside; Sop, sophorotrioside; Rha, rhamnoside; Gal, galactoside. The left and right block represent the log_2_Ratio(CK_40 DAH/0 DAH) and log_2_Ratio(Nar_40 DAH/0 DAH) of genes/metabolites, respectively. Metabolites labeled in red indicate the content of metabolites that significantly increased in Nar-treated fruit.

**Figure 6:**
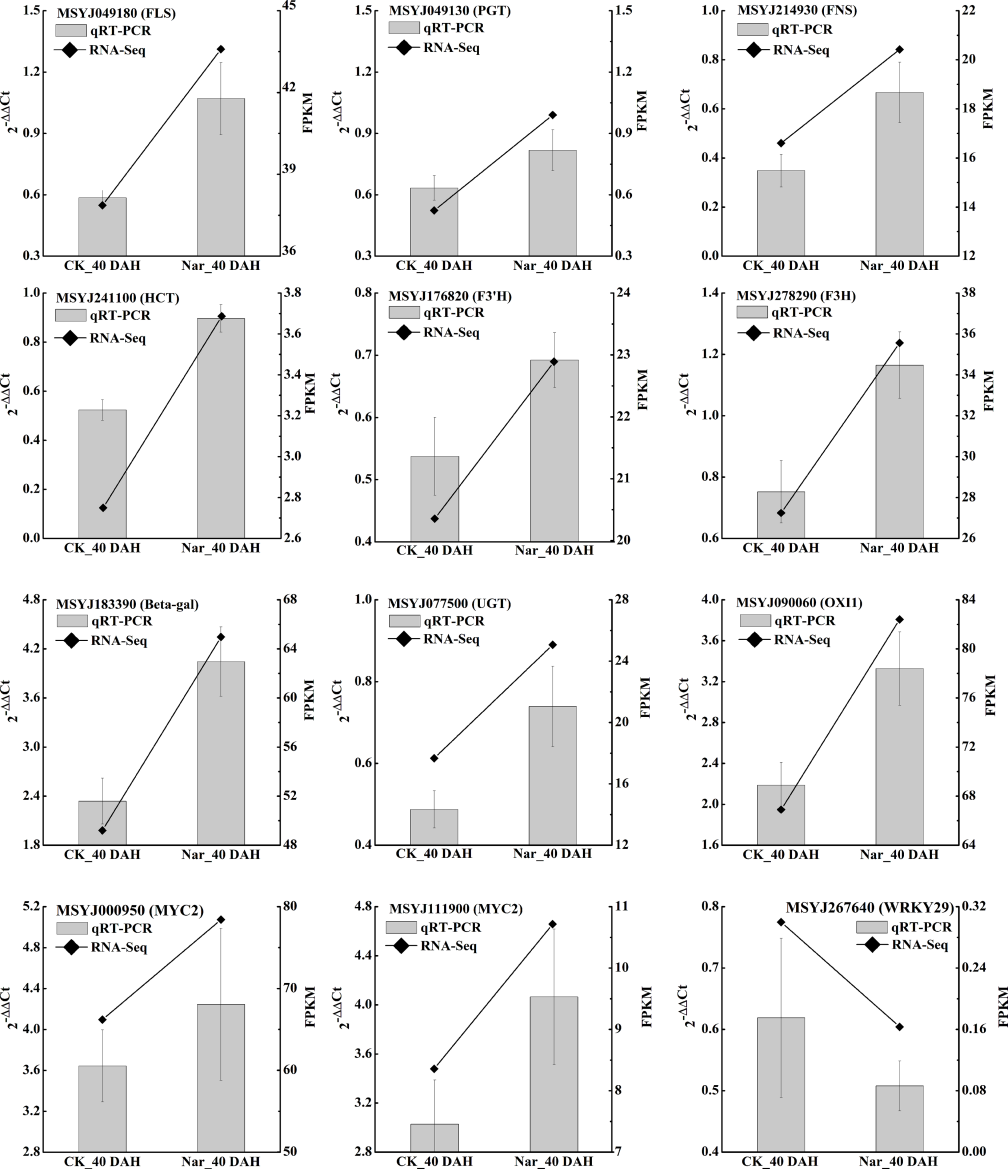
qRT-PCR validation of 12 citrus DEGs. Polylines show the FPKM value from RNA-Seq and bar charts show the qRT-PCR results of these genes, with each bar representing the mean ± standard error of three biological replicates. 0 DAH fruit was used as the calibration sample, *CsActin* was set as the internal reference gene for normalizing the transcript profiles following the 2^-△△Ct^ method.

Secondary metabolite biosynthesis was associated with significant enrichment in the KEGG enrichment analysis (**Figure 4F**), with phenylpropanoid and flavonoid biosynthesis accounting for much of the total. The phenylpropanoid and flavonoid metabolic pathways were analyzed to understand the changes occurring in terms of these processes during citrus fruit decay. Most flavones (Luteolin-7-O-(2’’’’-O-rhamnosyl)sophorotrioside, Luteolin-8-C-glucoside, etc.), flavanones (naringenin, Naringenin-7-O-Rutinoside-4’-O-glucoside, butin, etc.), and flavonols (Quercetin-3-O-rhamnoside, Kaempferol-3-O-rutinoside, etc.) were upregulated 40 d after treatment with naringin (**Figure 5**). According to the RNA-Seq results, naringin treatment also regulated the expression of pathway genes. The majority of DEGs in the Nar_40 DAH vs 0 DAH group exhibited both up- and down-regulation as compared to the CK_40 DAH vs 0 DAH group (**Figure 5**). Of these, five upstream genes (*CcHCT*, *CcPGT*, *CcFNS*, *CcF3H*, *CcF3’H*) and two downstream genes (*CcFLS*, *CcUGT*) showed up-regulated expression (**Figure 6**) in accordance with the content improvement of metabolites during citrus decay.

### Verification of gene expression through qRT-PCR

To validate the results of the transcriptomic analysis in the present study, we further selected 12 DEGs involved in significantly enriched phenylpropanoid biosynthesis, flavonoid biosynthesis, and the MAPK signaling pathway for study with qRT-PCR. The expression levels of most flavonoid biosynthesis genes and MYC in naringin-treated fruit were significantly higher than those in the control, while WRKY29 expression was downregulated under naringin treatment. Comparative analysis of all the selected genes obtained using qRT-PCR analysis showed similar expression patterns as those obtained by RNA-Seq (**Figure 6**), indicating the reliability of the expression data found using this method.

### Coexpression network of phenylpropanoid and flavonoid pathway genes

**Figure 7:**
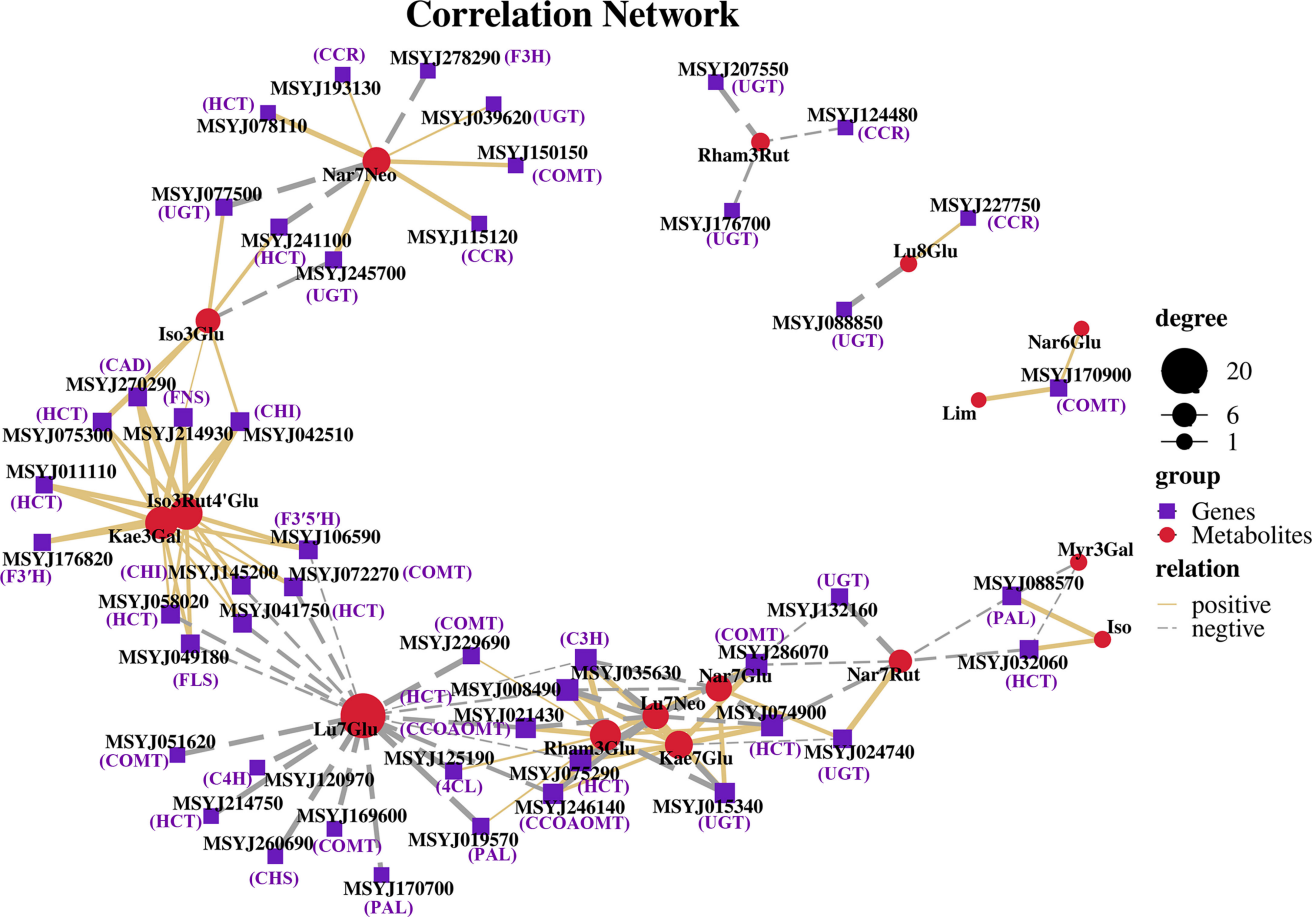
Coexpression networks between candidate genes and metabolites in phenylpropanoid and flavonoid biosynthesis pathways. Nodes represent genes or metabolites, purple font represent description of gene names, edges with solid lines and long dashes represent positive and negative correlation, and correlation is determined by a Pearson correlation coefficient of >0.5 or <-0.5, respectively.

Based on the DEGs and metabolite profile data, Pearson’s correlation coefficient analysis was conducted to calculate the degree of correlation between DEGs and DMs in the phenylpropanoid and flavonoid biosynthesis pathways. The results showed that 16 DMs were significantly associated with forty-seven DEGs. Specifically, 62 pairs were significantly and positively correlated (*rho* > 0.5), whereas 51 pairs presented significant and negative correlations (*rho* < -0.5) (**Figure 7**). Lu7Glu (cynaroside) was associated with the highest number of DEGS (20), and exhibited a significant and negative correlation with related genes, with followed by Rham3Glu a close second (Rhamnetin-3-O-Glucoside; 14DEGs), and Iso3Rut4’Glu (Isorhamnetin-3-O-rutinoside-4’-O-glucoside; 12 DEGs), Kae3Gal (Trifolin; 12 DEGs), and Iso (Isorhamnetin) demonstrating a highly positive correlation with most of their biosynthesis genes.

## Discussion

### Naringin treatment delays citrus decay and maintains quality

Senescence of postharvest citrus fruits is the main physiological action and is manifested as decay and quality deterioration, as associated with weight loss, changes in peel color, nutritional decline, and respiration rate enhancement. Numerous studies have demonstrated that exogenous flavonoids, including naringin, can effectively suppress disease and maintain the quality of postharvest fruits such as citrus (Ortuño and Río, 2009; Ortuño et al., 2011), litchi (Bai et al., 2022), and banana (Chen et al., 2019). Naringin supplement is easily extracted and has been widely applied in the health and cosmetics industries, although its inhibition of fungal (e.g., *P. digitatum*) growth is 25% and less than 75% for nobiletin and 38% for hesperidin (Ortuño et al., 2006; Salas et al., 2011). A similar result was obtained in this study, with delays in the decay rate of postharvest Nanfeng fruit observed as a result of naringin treatment, which was particularly noticeable after 30 d of storage (**Figure 1**). In addition, the total soluble solid and b* values were higher and lower in naringin-treated fruits, respectively, indicating quality maintenance and delayed color changes in these fruits.

### Naringin strengthens citrus fruit antioxidant capacity

The incidence of pathogen-related diseases, oxidative stress disorders, and senescence has increased dramatically (Ortuño and Río, 2009; Ballester et al., 2013). Antioxidant ability is a vital physiological mechanism for delaying fruit senescence and antifungal activity (Meitha et al., 2020). Bioactive compounds with antioxidant capacity extracted from natural sources have received considerable attention from researchers (Anwar et al., 2018), with previous studies revealing that flavonoids are naturally occurring antioxidants that function as signaling and defense molecules (Brodowska, 2017). The distribution and abundance of flavonoids can be affected by the oxidative stress that is generated by pathogenic infection or abiotic stress (Treutter, 2005; Nabavi et al., 2020). Naringin, a common flavanone, scavenges free radicals and decreases the oxidative stress that results from pathogenic processes during storage (Roghini and Vijayalakshmi, 2018). In the present study, naringin remarkably enhanced the total phenols, total flavonoids, and antioxidant activity of the stored citrus fruit, while suppressing the decay rate (**Figure 1**), suggesting that naringin treatment can inhibit the oxidative stress that is associated with senescence and disease invasion in citrus fruit.

Reducing oxidative species (ROS), including H_2_O_2_, OH^-^, and O_2_^-•^ etc., are reactive molecules activated in response to adverse conditions, such as pathogen invasion (Apel and Hirt, 2004). Antioxidant system that includes enzymatic components (SOD, POD, and CAT etc.), phenol and flavonoid components were reported to scavenging radical, and balancing ROS and antioxidant content (Apel and Hirt, 2004; Meitha et al., 2020). In the current study, naringin treatment enhanced the enzymatic activity of PAL, CAT, POD, and PPO in different storage period, accompanied by the inhibition of H_2_O_2_ content (**Figure 2**). Naringin appears to delay disease mainly because of the antioxidant system of components such as flavonoids (naringin) or enzymes (POD).

### Naringin promotes secondary metabolite accumulation

Metabolomics is an effective technique used for measuring the structure and function of plant metabolites modifications (Wang et al., 2019; Li et al., 2020). Untargeted and targeted metabolomic tools based on LC-MS or GC-MS analysis have also been developed to identify DMs in citrus species during development and storage period (Wang et al., 2016; Wang et al., 2017; Kim et al., 2022). In this study, an untargeted metabolomic approach was used to investigate the metabolic changes in freshly harvested (0 DAH) and stored citrus peel (CK_40 DAH and Nar_40 DAH) over 40 d. A total of 1010 metabolites from 10 classes were detected in the citrus peel. The flavonoid and coumarin contents in the phenylpropanoid pathway were significantly higher in the Nar_40 DAH group than the CK_40 DAH group, which is in agreement with previous studies showing that phenylpropanoid compounds, such as naringin and naringenin, can induce plant defenses against pathogens (Dixon et al., 1996; Dixon et al., 2002; Treutter, 2005). Other studies have also documented that coumarin derivatives, such as 5’- hydroxy-auraptene and phenolic hydroxyl moieties, show antioxidant and antifungal activities (Ali et al., 2021; Mustafa et al., 2021). This suggests that pathogen infection stimulates the accumulation of phenylpropenoid compounds during senescence and citrus infection, and that a large number of phenylpropenoid metabolites are synthesized following naringin treatment.

### Naringin activates the expression of phenylpropanoid pathway genes

The phenylpropanoid pathway refers to common flavonoid or lignin biosynthesis, as well as various other metabolites such as coumarins, stilbenes, or tannins (Vogt, 2010). Genes and gene products often form regulatory networks to perform necessary functions. Correlation analysis is widely used to describe the correlation patterns amongst genes and metabolites, and can be used to screen highly related genes in the phenylpropanoid pathway (Yuan et al., 2022). Abundant genes were matched and correlated significantly with their related metabolites, with both positive and negative correlations observed (**Figure 7**). Relatively few changes were observed in PAL, C4H, 4CL, and CHS, which are early biosynthetic genes in the phenylpropanoid pathway, excluding MSYJ088570. We also analyzed lignin biosynthetic genes and observed downregulation patterns following naringin treatment. This suggests a low correlation between lignin and citrus decay after 40 d of storage. Moreover, most late biosynthetic genes involved in flavonoid biosynthesis were upregulated. CHI was the first reported enzyme located upstream of the flavonoid biosynthetic pathway (Yin et al., 2019). Previous studies have shown that CHI plays a crucial role in plant responses to various pathogens (Zhou et al., 2018a). F3H, F3’H, and F3’5’H convert naringenin into flavones, flavonols, and flavanones (Liu et al., 2021), and their expression levels were upregulated in the Nar_40 DAH vs 0 DAH group, leading to competition for substrates. In the biosynthesis of flavonols and flavones, the UGTs family catalyzes glycosylation at the O-3 or O-7 position (Li et al., 2020; Liu et al., 2021). We observed that UGTs (MSYJ275760) were significantly upregulated in the Nar_40 DAH vs 0 DAH group, while MSYJ105070 and MSYJ128560 were downregulated after naringin treatment. Therefore, we inferred that the genes involved in phenylpropanoid compound biosynthesis could be important, and that the induction of these metabolite-related genes by naringin could increase the flavonoid content in infected citrus.

### DEGs in KEGG pathway

Expression pattern analysis of differential genes can be used to provide correlation with candidate gene functions at the initiated decay stages. In this study, KEGG pathway analysis identified four modules that are related to phenylpropanoid biosynthesis (ko00940), flavonoid biosynthesis (ko00941), stilbenoid, diarylheptanoid, and gingerol biosynthesis (ko00945), and the MAPK signaling pathway (ko04016), including phenylpropanoid compound biosynthesis genes and three transcription factors, MYC and WRKY. Reports have revealed that gene expression and secondary metabolite accumulation are induced in citrus fruits in response to *P. digitatum* infection (González-Candelas et al., 2010; Ballester et al., 2013). In addition to phenylpropanoid biosynthetic genes, transcriptional factors such as MYB, bHLH, ERF, and WRKY are also associated with the regulation of flavonoid biosynthesis and/or disease resistance in plants (Liu et al., 2021; Naik et al., 2022). WRKY transcription has been implicated in the regulation of flavonoid biosynthesis (Naik et al., 2022), and also acts as a transcriptional activator that participates in citrus disease resistance against *P. digitatum* (Wang et al., 2021; Wang et al., 2022). MYC, also known as the bHLH transcription factor, activates genes that participate in plant secondary metabolite biosynthesis and exhibits beneficial effects on plant growth *via* resisting fungal pathogens (Teng et al., 2021).

## Conclusion

In conclusion, this study demonstrated that exogenous naringin treatment maintained citrus fruit quality and delayed disease incidence; however, pathogens were not directly killed. After naringin treatment, antioxidant compounds such as phenols and flavonoids accumulated, antixodiant enzymes such as POD and PPO activities, and antioxidant capacity were enhanced. Differental metabolites and differentially expressed genes in phenylpropanoid and flavonoid biosynthesis pathway were enriched. The results suggest that secondary metabolites such as auraptene, butin, naringenin, and luteolin derivatives are potential antioxidant compounds that protect against pathogenic infection. Moreover, genes such as PGT, FNS, F3H, F3’H, FLS, and UGTs in the phenylpropanoid and flavonoid biosynthesis pathways, may be important factors that induce disease resistance in citrus fruits. Overall, our study revealed that several antioxidant-related secondary metabolites are induced by naringin treatment, which might play a major role in the plant’s response to pathogenic infection and in maintaining citrus fruit quality.

## Data availability statement

The data presented in the study are deposited in the National Center for Biotechnology Information (NCBI) repository, BioProject accession number PRJNA885437.

## Author contributions

JZ performed the experiments and wrote the main manuscript text. CC analyzed the data and provided advises. MC revised the manuscript and assistance to the submission of the final manuscript. JC designed the research and provided advises. All authors reviewed the manuscript and approved the submitted version.

## Funding

This work was supported by the Two Thousand Plan Foundation of Jiangxi Province (jxsq2020101076) and the Priming Scientific Research Foundation of Jiangxi Agricultural University (9232309106).

## Conflict of interest

The authors declare that the research was conducted in the absence of any commercial or financial relationships that could be construed as a potential conflict of interest.

## Publisher’s note

All claims expressed in this article are solely those of the authors and do not necessarily represent those of their affiliated organizations, or those of the publisher, the editors and the reviewers. Any product that may be evaluated in this article, or claim that may be made by its manufacturer, is not guaranteed or endorsed by the publisher.
