# Supplementary material for: Comparative transcriptomic and metabolomic analyses reveal the delaying effect of naringin on postharvest decay in citrus fruit
Source: Front Plant Sci. 2022 Nov 30;13:1045857. doi: 10.3389/fpls.2022.1045857 (PMC9748555; doi:10.3389/fpls.2022.1045857)
Supplement: Supplementary file 1 [file DataSheet_1.zip › Data Sheet 1/Table S1.docx]

**Supplementary Table S1:** Primers used for qRT-PCR analysis.

| Gene ID | Forward primer | Reverse primer | Gene description |
| --- | --- | --- | --- |
| MSYJ049180 | TCGTGGCCAGTTTTCTTGGA | AGATCAAACATGAGCGGCCA | Q3-FLS |
| MSYJ049130 | ATGATGGTGGCTCGTCGTTT | AGTGGGTAGAGGTGTATTTTGCT | phlorizin synthase |
| MSYJ214930 | TGGAACTGGGAGAAGAGGCT | TCCTGTGCTCTTGGAGATGC | flavone synthase II |
| MSYJ241100 | TATGTGGGTTGCTTGGGGTC | ATGGGGCTAGCTAAGGGGAA | shikimate O-hydroxycinnamoyltransferase |
| MSYJ176820 | GACTGCAACATTGGCCCATG | ACGGGCCCTTAATTTCCTCA | flavonoid 3'-monooxygenase |
| MSYJ278290 | AAGACCTTGAACTCGCCAGG | AAGAAGCAACGAAGGGTAGGG | naringenin 3-dioxygenase |
| MSYJ183390 | ACCCGTCAATGCTCTTGGAG | CATTCCGTCCACTGTAGCCG | beta-glucosidase |
| MSYJ077500 | CAAAGAAGGGCACTCATCGA | CACCTTGACCCAGCGTTACT | coniferyl-alcohol glucosyltransferase |
| MSYJ090060 | ATGAGAGCGATGGTAGGGGT | TCCTCTTCACAAGCATGACGT | serine/threonine-protein kinase OXI1 |
| MSYJ000950 | TAATGCAAGCCTTGAAAGAGTTGG | CAACTTTGGCTGCAAGGACG | transcription factor MYC2 |
| MSYJ111900 | TGCTTCAAGATATTGTGGTTAGGG | GCATCCAAAGAACACAACTCGT | transcription factor MYC2 |
| MSYJ267640 | TGCGTTCAAAGATGGCCAAG | ACCAAGAGTTGTCATCCGCA | WRKY transcription factor 29 |
| XM_006464053 | CATCCCTCAGCACCTTCC | CCAACCTTAGCACTTCTCC | CsActin-7 |
