# Supplementary material for: Comparative transcriptomic and metabolomic analyses reveal the delaying effect of naringin on postharvest decay in citrus fruit
Source: Front Plant Sci. 2022 Nov 30;13:1045857. doi: 10.3389/fpls.2022.1045857 (PMC9748555; doi:10.3389/fpls.2022.1045857)
Supplement: Supplementary file 1 [file DataSheet_1.zip › Data Sheet 1/Table S2.docx]

**Supplementary Table S2:** Impact of naringin treatment on respiration, weight, color, TSS, TA, and VC during citrus postharvest storage.

| Attribute | Treatment | Stage  0 DAH | 10 DAH | 20 DAH | 30 DAH | 40 DAH | 50 DAH | 60 DAH |
| --- | --- | --- | --- | --- | --- | --- | --- | --- |
| Respiration rate  (mgCO_2_/kg(FW)/h) | Control | 39.795±0.532 | 27.573±0.236 | 15.811±0.825 | 20.726±2.099 | 19.557±0.470 | 22.484±2.463 | 23.311±2.227 |
|  | Naringin | 39.795±0.532 | 23.411±0.830 | 14.820±0.296 | 19.248±1.004 | 20.933±1.060 | 20.258±0.692 | 21.803±2.393 |
| Weight loss (%) | Control | 0 | 1.592±0.048 | 2.441±0.036 | 3.541±0.048 | 4.507±0.030 | 5.545±0.268 | 6.361±0.316 |
|  | Naringin | 0 | 1.613±0.055 | 2.497±0.043 | 3.491±0.035 | 4.472±0.067 | 5.428±0.063 | 6.112±0.055 |
| *L ** | Control | 66.759±0.200 | 65.407±0.290 | 64.769±0.407 | 63.469±0.322 | 63.308±0.256 | 61.913±0.258 | 62.149±0.243 |
|  | Naringin | 66.759±0.200 | 65.621±0.287 | 64.721±0.344 | 63.368±0.313 | 63.086±0.372 | 62.469±0.335 | 62.292±0.326 |
| *a** | Control | 16.272±0.790 | 20.568±0.766 | 27.435±0.841 | 30.476±0.584 | 31.236±0.450 | 33.699±0.507 | 34.132±0.429 |
|  | Naringin | 16.272±0.790 | 19.723±0.999 | 26.319±0.835 | 27.456±0.648 | 30.512±0.609 | 31.242±0.412 | 32.862±0.473 |
| *b** | Control | 66.727±0.417 | 63.292±0.324 | 63.512±0.351 | 62.829±0.489 | 62.173±0.393 | 61.621±0.376 | 62.754±0.435 |
|  | Naringin | 66.727±0.417 | 63.269±0.295 | 62.954±0.404 | 62.352±0.525 | 60.955±0.557 | 59.712±0.519 | 61.628±0.409 |
| TSS (%) | Control | 12.570±0.063 | 13.980±0.042 | 13.310±0.035 | 13.620±0.013 | 13.600±0.015 | 13.600±0.021 | 12.930±0.015 |
|  | Naringin | 12.570±0.063 | 13.800±0.158 | 13.930±0.030 | 14.190±0.010 | 13.530±0.056 | 13.850±0.031 | 14.560±0.027 |
| TA (%) | Control | 0.533±0.017 | 0.620±0.014 | 0.593±0.012 | 0.520±0.010 | 0.493±0.008 | 0.540±0.014 | 0.367±0.016 |
|  | Naringin | 0.533±0.017 | 0.533±0.008 | 0.500±0.014 | 0.600±0.015 | 0.460±0.009 | 0.487±0.016 | 0.467±0.013 |
| VC (mg/100g FW) | Control | 20.290±1.028 | 17.210±0.362 | 17.482±0.327 | 16.757±0.091 | 15.217±0.140 | 15.217±0.306 | 12.772±0.186 |
|  | Naringin | 20.290±1.028 | 18.659±0.389 | 17.301±0.259 | 17.482±0.218 | 14.040±0.167 | 14.855±0.181 | 16.757±0.091 |
|  |  |  |  |  |  |  |  |  |
| Significance ^b^  Attributes | Respiration rate | Weight loss | *L** | *a** | *b** | TSS | TA | VC |
| Storage time | ** | ** | ** | ** | ** | ** | ** | ** |
| Treatment | Ns | Ns | Ns | ** | ** | ** | Ns | * |
| Storage time×Treatment | Ns | Ns | Ns | Ns | Ns | ** | ** | ** |

^b^ Ns, *, **, no significant or significant at 0.05 or 0.01 level, respectively. The multi-comparison correlation was analyzed using Duncan’s multiple range test (5 % level) in the PROC ANOVA.
