# Supplementary figures and images for: Comparative transcriptomic and metabolomic analyses reveal the delaying effect of naringin on postharvest decay in citrus fruit
Source: Front Plant Sci. 2022 Nov 30;13:1045857. doi: 10.3389/fpls.2022.1045857 (PMC9748555; doi:10.3389/fpls.2022.1045857)

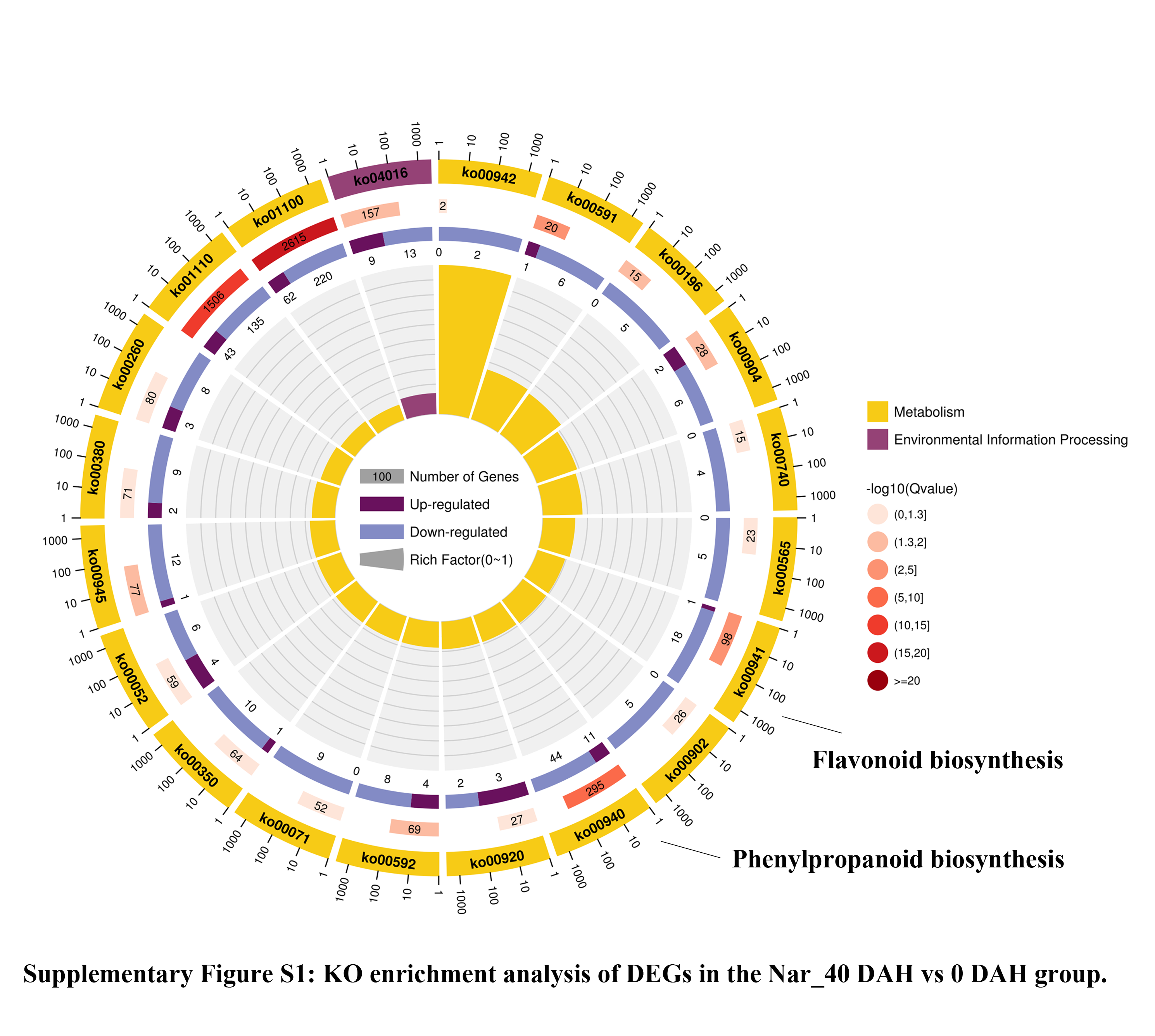

Supplement: Supplementary file 1 [file DataSheet_1.zip › Data Sheet 1/Figure S1.tif]
